# Infant mortality trend in the city of Rio Branco, AC, 1999 to 2015

**DOI:** 10.11606/S1518-8787.2018052000280

**Published:** 2018-03-14

**Authors:** Alanderson Alves Ramalho, Andréia Moreira de Andrade, Fernanda Andrade Martins, Rosalina Jorge Koifman

**Affiliations:** I Universidade Federal do Acre. Centro de Ciências da Saúde e do Desporto. Rio Branco, AC, Brasil; II Fundação Oswaldo Cruz. Escola Nacional de Saúde Pública. Rio de Janeiro, RJ, Brasil

**Keywords:** Infant Mortality, trends, Cause of Death, Quality of Health Care, Health Status Indicators, Time Series Studies, Mortalidade Infantil, tendências, Causas de Morte, Qualidade da Assistência à Saúde, Indicadores Básicos de Saúde, Estudos de Séries Temporais

## Abstract

**OBJECTIVE:**

Analyze the trend of infant mortality in Rio Branco, state of Acre, from 1999 to 2015.

**METHODS:**

An ecological observational study of a time series, in which data from deaths from the Information System on Mortality and Births of the Information System on Live Births were used. The annual percentage change was estimated using the Joinpoint software.

**RESULTS:**

The infant mortality rate decreased from 26.99 in 1999 to 14.50 in 2015 per 1,000 live births, with an annual percentage change of -4.37 (95%CI -5.4– -3.4). When stratified by age components, the neonatal period presented an annual percentage change of -4.73 (95%CI -5.7– -3.7), and the post-neonatal period was -3.7 (95%CI -5.4– -2.0). Avoidability, avoidable causes and poorly defined causes showed a downward trend throughout the period and causes not clearly preventable showed an upward trend until 2008. The group of causes that contributed most to the infant deaths during the period studied was perinatal diseases, followed by malformations, infectious and parasitic diseases, and respiratory diseases.

**CONCLUSIONS:**

Despite the decreasing trend in infant mortality rates in the capital compared to developed countries, it is relatively high.

## RESUMO

**OBJETIVO:**

Analisar a tendência da mortalidade infantil em Rio Branco, AC, de 1999 a 2015.

**MÉTODOS:**

Estudo observacional ecológico de série temporal, em que foram utilizados dados de óbitos do Sistema de Informações sobre Mortalidade e Nascimentos do Sistema de Informações sobre Nascidos Vivos. Estimou-se a variação percentual anual por meio do *software* Joinpoint.

**RESULTADOS:**

A taxa de mortalidade infantil reduziu de 26,99, em 1999, para 14,50, em 2015, por 1.000 nascidos vivos, com variação percentual anual de -4,37 (IC95% -5,4– -3,4). Quando estratificada por componentes etários, o neonatal apresentou variação percentual anual de -4,73 (IC95% -5,7– -3,7), e o pós-neonatal de -3,7 (IC95% -5,4– -2,0). A evitabilidade, as causas evitáveis e as causas mal definidas apresentaram tendência descendente em todo o período e as causas não claramente evitáveis apresentaram tendência ascendente até 2008. O grupo de causas que mais contribuiu para os óbitos infantis no período estudado foi o das afecções perinatais, seguido pelo das malformações, das doenças infecciosas e parasitárias, e do grupo das doenças respiratórias.

**CONCLUSÕES:**

Apesar da tendência decrescente da taxa de mortalidade infantil na capital, quando comparada à de países desenvolvidos, mostra-se relativamente elevada.

## INTRODUCTION

The conditions of life and health of a population can be evaluated through several health indicators. The infant mortality rate (IMR) is considered one of the most sensitive indicators of detecting changes. The quest for the reduction of IMR in underdeveloped and developing countries is part of the global government agenda and represents a major challenge for health services and society as a whole^1^.

Mortality of children younger than 1-year-old is commonly classified according to its components: neonatal (deaths from birth to the 27th day of life) and post-neonatal (deaths from 28th to 364th day of life).

Neonatal mortality is sensitive to endogenous or biological factors related to gestation and delivery. Its reduction involves greater complexity and greater cost in the prevention of these deaths related to genetic problems, fetal malformation, late pregnancy and complications in childbirth and postpartum^2–4^.

Post-neonatal mortality is an indicator sensitive to external factors that influence the occurrence of deaths in this age group. They reflect the environment, nutritional and well-being conditions in which this population is inserted. In this component of the IMR, government actions, such as basic sanitation, income distribution, and a greater supply of medical services, have a greater impact on their reduction, especially when they include less favored social classes^5^ ^,^ ^6^.

The estimated infant mortality rate showed a declining trend worldwide, presenting a reduction of 49%, from 23 to 10 deaths per 1,000 live births from 1990 to 2015^7^. In recent years, Europe has had the largest reduction (55%), followed by the Americas (54%). The smallest reduction (21%) was observed in African countries.

In South America, considering the ranking of the best IMR estimates for 2015, Brazil is behind Chile, Uruguay, Argentina, Venezuela, Peru and Colombia; and ahead of Ecuador, Paraguay, Suriname, Bolivia, and Guyana^7^. The Ministry of Health analyzed the IMR trend in Brazil from 1998 to 2007 and observed a decline in all regions^8^. At the end of the analyzed period, the highest rates were recorded in the Northeast region (28.7/1,000 live births [LB]), followed by the North (22.1/1,000 LB), Midwest (16.5/1,000 LB), Southeast (14.6/1,000 LB), and South (12.9/1,000 LB). This arrangement was unchanged during this time series, indicating the maintenance of the regional differences existing in the country^1^.

Knowing the evolution of IMR can favor decision making in the conduct of public health policies to ensure better assistance during prenatal, childbirth and puerperium, efforts needed to reduce infant mortality. There is a shortage of publications that used time series analysis of infant mortality in the northern region of Brazil. In the state of Acre, no studies with this methodology were found. Thus, the objective of this study was to analyze the trend of infant mortality in Rio Branco, Brazil, from 1999 to 2015.

## METHODS

Observational ecological descriptive study of a time series of infant mortality in Rio Branco between 1999 and 2015. Rio Branco, capital of Acre, has 348,354 inhabitants (45.9% of the state population), distributed over an area of 9,962 km^2^ (6.5% of the territory of the state), and about 90% reside in the urban area^9^.

The data sources were the vital statistics of the Department of Informatics of the National Health System (DATASUS). The number of deaths of children under one year of age was obtained from the Mortality Information System (SIM) and the number of births from the Information System on Live Births (SINASC).

The IMR were calculated by three categorization systems: age components, avoidability and according to the main groups of basic cause of death.

The IMR was defined as the ratio between the total number of deaths in children under one year of age in the year and in the denominator, the total number of live births expressed per 1,000 live births. The early neonatal component was obtained by the ratio of the number of deaths between zero and six days of life recorded in the year divided by the total number of live births. The late neonatal component corresponded to this same ratio, with the number of deaths from seven to 27 days in the numerator and the post-neonatal component, the number of deaths from 28 to 364 days.

For the classification by avoidability, the Brazilian List of Avoidable Deaths^10^ was used, which considers causes of ill-defined deaths, avoidable causes (reducible by immunization actions, reducible by adequate attention to women during pregnancy and delivery and to the newborn, reducible by appropriate diagnostic and treatment actions, and reducible by health promotion actions linked to health care actions); and non-preventable causes (other causes).

The cause of death registry was based on the 10th International Statistical Classification of Diseases and Related Health Problems (ICD-10). The following groups were defined for the analysis of causes of death: perinatal conditions (ICD-10 P00 to P96); malformations, deformities and abnormalities (ICD-10 Q00 to Q99); respiratory diseases (ICD-10 J00 to J99); infectious and parasitic diseases (ICD-10 A00 to B99); and other causes (all other ICD-10 codes). In this categorization, deaths due to poorly defined causes in children under one year were redistributed proportionately among the groups of causes analyzed.

The trend analysis was performed using estimates of annual percentage change (APC) and the average annual percentage change (AAPC) of the infant mortality rate from 1999 to 2015 by means of Poisson regression. The statistical program Joinpoint (http://surveillance.cancer.gov/joinpoint/) was used. The joinpoint technique uses the log-transformed rates to identify the joinpoints over the period that are able to describe a significant change in the trend through APC^11^. Because biological phenomena do not always behave uniformly, a rate may exhibit changes in the variation rate over time. When this situation occurs, the analysis of segments may better represent the phenomenon observed. In the segment APC concept (APC), the joinpoints correspond to k-1 segments. The summary measure of the various APC is the AAPC which corresponds to the average annual percentage change. In situations where only one APC comprises the entire period studied, the AAPC corresponds to the APC. The tests of significance to choose the best model were based on the Monte Carlo permutation method, considering p < 0.05. To minimize the effect of possible autocorrelation, the option “fit an autocorrelated errors model based on the data” was used in the software.

## RESULTS

A total of 123,800 children were born alive in Rio Branco from 1999 to 2015. The annual average was 7,282.35 (SD = 624.27, min. = 6,437, max. = 8,819). The live births trend presented an annual percentage change of -2.29 (95%CI -2.9– -1.7) by 2010. Subsequently, the annual percentage change was 1.64, however, there was no statistical evidence for the variation from 2010 to 2015 (95%CI -0.5–3.8).

The precocious neonatal component contributed the most to infant mortality in the period, with an average proportion of 52.2% (max. = 62.2% in 1999, min. = 41.4% in 2009); the late neonatal component presented an average of 14.3% (max. = 19.8% in 2009, min. = 8.9% in 2012); and the post-neonatal component presented a mean of 33.5% (max. = 37.8% in 2006, min. = 25.6% in 1999). Regarding avoidability, avoidable causes contributed an average of 66.2% (min. = 57.6%, max. = 72.4%), those not clearly avoidable averaged 27.2% (min. = 14.2%, max. = 37.6%), and the ill-defined causes were 6.6% (min. = 0.9%, max. = 13.4%) (Table 1).

**Table 1:** Infant mortality rate per 1,000 live births and proportional mortality in children under one year of age (%) by age group, avoidance, and causes of death. Rio Branco, state of Acre, Brazil, 1999 to 2015.

Year	Absolute frequency		Infant mortality rate per thousand live births and proportional mortality (%)												

			Age group					Avoidability			Causes group				
			
	Live births	Infant deaths	< 1 year	Neonatal	EN	LN	PN	AC	NCA	PD	PC	MDA	RD	IPD	OC
1999	8,819	238	26.99	20.07	16.78 (62.2%)	3.29 (12.2%)	6.92 (25.6%)	18.82 (69.7%)	5.22 (19.3%)	2.95 (10.9%)	17.44 (64.6%)	3.95 (14.6%)	1.27 (4.7%)	1.53 (5.7%)	2.8 (10.4%)
2000	8,287	232	28	18.22	14.84 (53.0%)	3.38 (12.1%)	9.77 (34.9%)	20.27 (72.4%)	3.98 (14.2%)	3.74 (13.4%)	19.36 (69.2%)	3.06 (10.9%)	1.67 (6.0%)	1.95 (7.0%)	1.95 (7.0%)
2001	7,788	184	23.63	16.05	13.87 (58.7%)	2.18 (9.2%)	7.58 (32.1%)	16.95 (71.7%)	4.88 (20.7%)	1.8 (7.6%)	15.29 (64.7%)	3.89 (16.5%)	1.39 (5.9%)	1.81 (7.6%)	1.25 (5.3%)
2002	7,710	161	20.88	14.79	12.06 (57.8%)	2.72 (13.0%)	6.10 (29.2%)	14.66 (70.2%)	4.41 (21.1%	1.82 (8.7%)	13.78 (66.0%)	3.41 (16.3%)	0.57 (2.7%)	1.28 (6.1%)	1.85 (8.8%)
2003	7,669	171	22.3	15.52	12.52 (56.1%)	3.00 (13.5%)	6.78 (30.4%)	15.39 (69.0%)	5.09 (22.8%)	1.83 (8.2%)	13.63 (61.1%)	3.83 (17.2%)	0.85 (3.8%)	1.7 (7.6%)	2.27 (10.2%)
2004	7,259	164	22.59	14.74	12.12 (53.7%)	2.62 (11.6%)	7.85 (34.8%)	14.19 (62.8%)	5.37 (23.8%)	3.03 (13.4%)	14.00 (62.0%)	4.77 (21.1%)	1.27 (5.6%)	1.27 (5.6%)	1.27 (5.6%)
2005	7,288	149	20.44	12.49	10.29 (50.3%)	2.20 (10.7%)	7.96 (38.9%)	12.62 (61.7%)	6.45 (31.5%)	1.37 (6.7%)	10.74 (52.5%)	4.71 (23.0%)	1.03 (5.0%)	2.21 (10.8%)	1.76 (8.6%)
2006	7,345	135	18.38	11.44	9.39 (51.1%)	2.04 (11.1%)	6.94 (37.8%)	12.8 (69.6%)	5.17 (28.1%)	0.41 (2.2%)	9.33 (50.8%)	3.34 (18.2%)	0.97 (5.3%)	2.23 (12.1%)	2.51 (13.6%)
2007	7,067	155	21.93	14.57	11.89 (54.2%)	2.69 (12.3%)	7.36 (33.5%)	13.58 (61.9%)	7.5 (34.2%)	0.85 (3.9%)	10.75 (49.0%)	5.59 (25.5%)	1.62 (7.4%)	1.03 (4.7%)	2.94 (13.4%)
2008	7,068	125	17.69	11.18	8.21 (46.4%)	2.97 (16.8%)	6.51 (36.8%)	10.19 (57.6%)	6.51 (36.8%)	0.99 (5.6%)	8.09 (45.8%)	4.5 (25.4%)	0.6 (3.4%)	1.35 (7.6%)	3.15 (17.8%)
2009	6,538	116	17.74	10.86	7.34 (41.7%)	3.52 (20.0%)	6.73 (38.3%)	11.47 (64.7%)	5.66 (31.9%)	0.61 (3.4%)	9.19 (51.8%)	2.85 (16.1%)	1.11 (6.3%)	1.27 (7.1%)	3.33 (18.8%)
2010	6,437	117	18.18	11.96	8.54 (47.0%)	3.42 (18.8%)	6.21 (34.2%)	11.19 (61.5%)	5.13 (28.2%)	1.86 (10.3%)	10.91 (60.0%)	3.29 (18.1%)	0.52 (2.9%)	1.38 (7.6%)	2.08 (11.4%)
2011	6,902	90	13.04	8.98	7.53 (57.8%)	1.45 (11.1%)	4.06 (31.1%)	8.98 (68.9%)	3.33 (25.6%)	0.72 (5.6%)	8.13 (62.4%)	1.84 (14.1%)	0.77 (5.9%)	0.77 (5.9%)	1.53 (11.8%)
2012	6,531	79	12.1	7.96	6.89 (57.0%)	1.07 (8.9%)	4.13 (34.2%)	7.81 (64.6%)	3.22 (26.6%)	1.07 (8.9%)	6.89 (56.9%)	2.69 (22.2%)	0.84 (6.9%)	0.50 (4.2%)	1.18 (9.7%)
2013	7,116	95	13.35	9.42	6.89 (51.6%)	2.53 (18.9%)	3.93 (29.5%)	9.56 (71.6%)	3.51 (26.3%)	0.28 (2.1%)	8.33 (62.4%)	2.15 (16.1%)	0.57 (4.3%)	0.57 (4.3%)	1.72 (12.9%)
2014	7,011	107	15.26	9.84	6.56 (43.0%)	3.28 (21.5%)	5.42 (35.5%)	10.13 (66.4%)	4.99 (32.7%)	0.14 (0.9%)	7.77 (50.9%)	2.88 (18.9%)	1.15 (7.5%)	1.15 (7.5%)	2.30 (15.1%)
2015	6,965	101	14.50	9.76	6.60 (45.5%)	3.16 (21.8%)	4.74 (32.7%)	8.9 (61.4%)	5.46 (37.6%)	0.14 (1.0%)	7.68 (53.0%)	3.33 (23.0%)	1.01 (7.0%	0.87 (6.0%)	1.59 (11.0%)

EN: early neonatal; LN: late neonatal; PN: post-neonatal; AC: avoidable causes; NCA: not clearly avoidable; PD: poorly defined; PC: perinatal conditions; MDA: malformations, deformities, and abnormalities; RD: respiratory diseases; IPD: infectious and parasitic diseases; OC: other causes

The group of basic causes that contributed the most to infant deaths during the period was perinatal conditions (mean = 57.8%, min. = 45.8%, max. = 69.2%), followed by malformations (mean = 18.7%, min. = 10.9%, max. = 25.5%), infectious and parasitic diseases (mean = 6.9%, min. = 4.2%, max. = 12.2%), and respiratory diseases (mean = 5.3%, min. = 2.7%, max. = 7.4%). The other grouped causes contributed, on average, to 11.3% of infant deaths (min. = 5.3%, max. = 18.8%) (Table 1).

**Figure 1:**
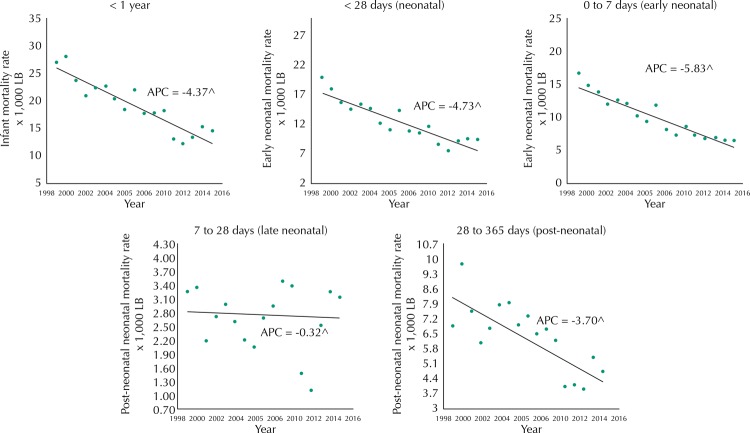
The trend in infant mortality rates by age groups. Rio Branco, state of Acre, Brazil, 1999 to 2015.

APC: annual percentage change; LB: live births

^ statistically different from zero.

The IMR (per 1,000 live births) was reduced in the analyzed period from 27.0 in 1999 to 14.5 in 2015. This reduction also occurred when stratified by components (early and late neonatal and post-neonatal), avoidability criteria, and groups of causes (Table 1). This significant reduction in IMR between 1999 and 2015 had an APC of -4.4 (95%CI -5.4– -3.4) (Figure 1, Table 2).

**Table 2:** Distribution of the annual percentage change (APC) of infant mortality rates by age group, avoidability, and causes of death. Rio Branco, state of Acre, Brazil, 1999 to 2015.

Distribution	Mortality rate		Deaths	VPA (95%CI)	Period

	1999	2015			
< 1 year	26.99	14.50	2,419	-4.37 (-5.4– -3.4)	1999–2015
Age group					
Neonatal	20.07	9.76	1,616	-4.73 (-5.7– -3.7)	1999–2015
EN	16.78	6.60	1,283	-5.83 (-6.7– -4.9)	1999–2015
LN	3.29	3.16	333	-0.32 (-3.0–2.4)	1999–2015
Post-neonatal	6.92	4.74	802	-3.7 (-5.4– -2.0)	1999–2015
Avoidability					
AC	18.82	8.90	1,615	-5.04 (-6.0– -4.1)	1999–2015
NCA	5.22	5.46	625	5.18 (1.2–9.3)	1999–2008
				-17.86 (-35.3–4.4)	2008–2012
				21.75 (-4.0–54.5)	2012–2015
PD	2.95	0.14	179	-10.77 (-15.5– -5.7)	1999–2015
Causes group					
PC	17.44	7.68	1,314	-5.79 (-7.0– -4.6)	1999–2015
MDA	3.95	3.33	409	4.98 (-0.9–11.2)	1999–2007
				-18.33 (-37.1–6.0)	2007–2011
				9.30 (-9.5–32.0)	2011–2015
RD	1.27	1.01	118	-2.83 ( -6.0–0.4)	1999–2015
IPD	1.53	0.87	157	-4.79 (-8.0– -1.5)	1999–2015
OC	2.80	1.59	242	-27.47 (-66.2–55.8)	1999–2001
				10.64 (0.1–22.3)	2001–2009
				-26.82 (-69.4–75.2)	2009–2012
				18.16 (-24.7–85.4)	2012–2015

EN: early neonatal; LN: late neonatal; PN: post-neonatal; AC: avoidable causes; NCA: not clearly avoidable; PD: poorly defined; PC: perinatal conditions; MDA: malformations, deformities, and abnormalities; RD: respiratory diseases; IPD: infectious and parasitic diseases; OC: other causes

The neonatal component presented an APC of -4.7 (95%CI -5.7– -3.7). When it was classified as premature and late, the reduction occurred to the early neonatal component (APC = -5.8, 95%CI -6.7– -4.9), and there was insufficient statistical evidence to state variation in the late component. Post-neonatal mortality presented a significant downward trend with APC of -3.7 (95%CI -5.4– -2.0).

**Figure 2:**
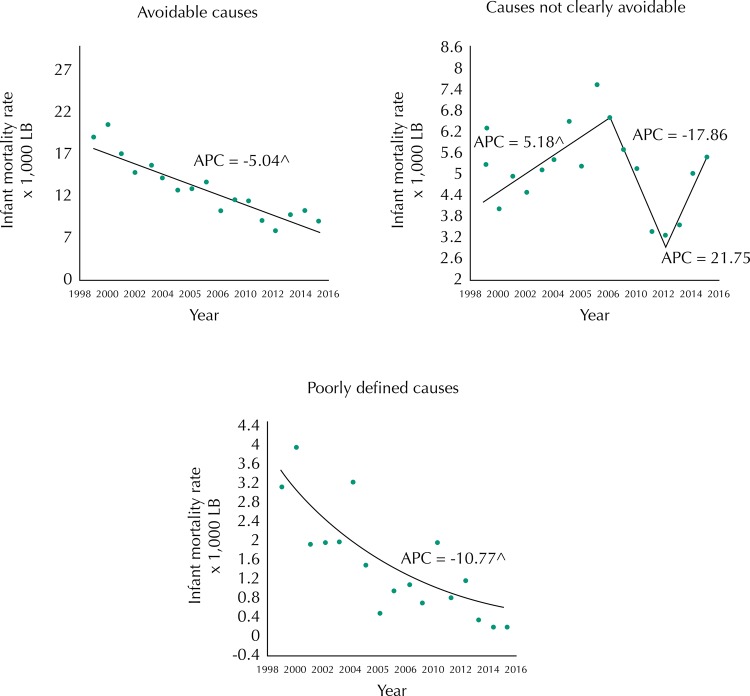
Trends in infant mortality rates due to avoidability and poorly-defined causes. Rio Branco, state of Acre, Brazil, 1999 to 2015.

APC: annual percentage change; LB: live births

^ statistically different from zero.

Infant mortality due to avoidance (Figure 2, Table 2) showed a downward trend for the entire period in the avoidable causes stratum, with an APC of -5.0 (95%CI -6.0– -4.1). The non-clearly avoidable causes showed an upward trend until 2009 with an APC of 5.2 (95%CI 1.2–9.3).

**Figure 3:**
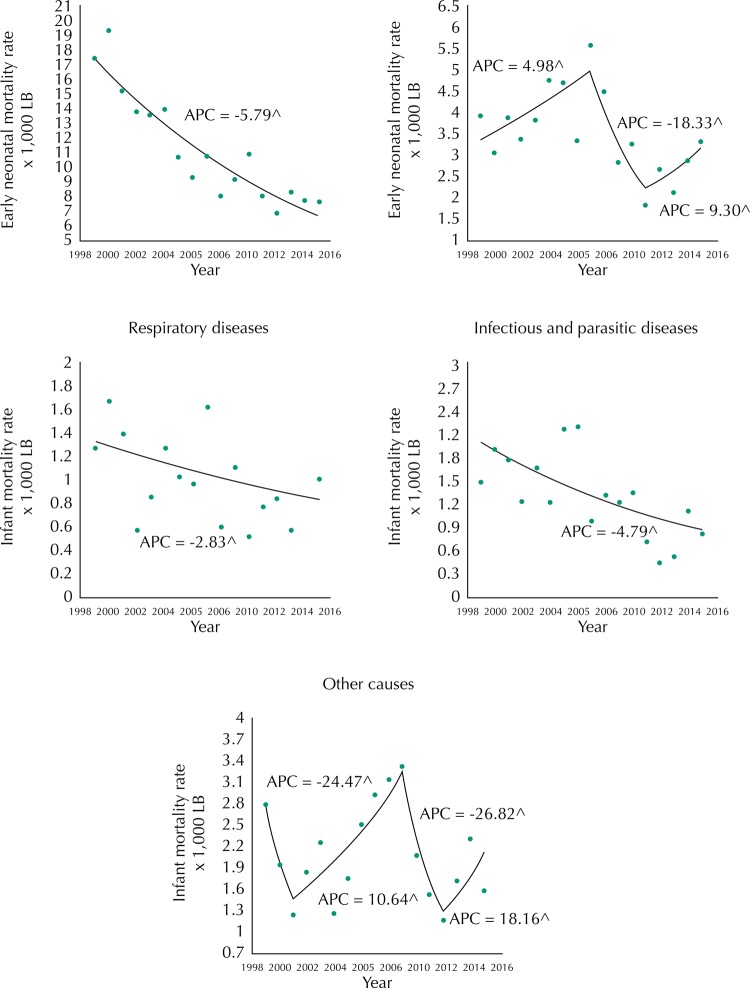
The trend in infant mortality rates by groups of causes. Rio Branco, state of Acre, Brazil, 1999 to 2015.

APC: annual percentage change; LB: live births

^ statistically different from zero.

The poorly defined causes presented a downward trend with APC of -10.8 (95%CI -15.5– -5.7). Perinatal conditions had an APC of -5.8 (95%CI -7.0– -4.6); respiratory diseases had an APC of -2.8 (95%CI -6.0–0.4) (Figure 3, Table 2). The malformations, deformities, and anomalies presented cyclical variations throughout the period and it was not possible to observe a trend with statistical significance.

Infectious diseases presented a decreasing trend during the whole period with APC of -4.8 (95%CI -8.0– -1.5). The other causes of death presented an upward trend from 2001 to 2009, with APC of 10.64 (95%CI 0.1–22.3). There was not enough statistical evidence to affirm annual percentage variation in the other periods (Figure 3, Table 2).

## DISCUSSION

In this study, the infant mortality rate decreased significantly in the period studied. When stratified by age components, the neonatal period presented an annual percentage change of -4.73 (95%CI -5.7– -3.7) and the post-neonatal period, -3.7 (95%CI -5.4– - 2.0). The avoidable causes and the ill-defined causes showed a downward trend throughout the period and the causes not clearly avoidable presented an upward trend until 2008. The group of causes that contributed the most to the infant deaths during the period studied was perinatal diseases, followed by malformations, infectious and parasitic diseases, and respiratory diseases.

The significant worldwide reduction in the magnitude of infant mortality occurred in an important way in Latin America and the Caribbean, according to the United Nations (UN) report in 2015, with an estimated decrease of 65.2% (in 1990, 46/1,000 LB; in 2015 of 16/1,000 LB). In developed countries, IMR decreased from 13 to six deaths per 1,000 live births in the same period^7^.

Rio Branco followed this trend of the world scenario, with a cumulative reduction in IMR of 46.3% from 1999 to 2015. The IMR (14.5/1,000 LB) in the Acre capital was lower than the national average (15.02/1,000 LB) in 2015. In Brazil, between 1990 and 2007, IMR fell from 47.1/1,000 LB in 1990 to 20.0/1,000 LB in 2007, with an APC of -5.1. The decline was slightly faster in the 1990s (APC = -5.5), and then the APC declined to -4.4 in the 2000s^12^.

The decrease in IMR occurred in all Brazilian regions, with the most notable changes in the Northeast, where the APC was -5.9 (IMR = 75.8/1,000 LV in 1990 to 28.7/1,000 LB in 2007). The declines in the North with APC of -4.3, Southeast (-4.9), South (-4.5) and Midwest (-4.1) were also substantial^12^.

Although the IMR in Brazil is high when compared to other countries with a similar economic development index^13^, it was enough for Brazil to achieve the fourth objective outlined in the document “Millennium Development Goals”^14^. The commitment was to reduce the national infant mortality rate to 15.7/1,000 LB by 2015.

When the infant mortality rate is very low, it is almost totally represented by neonatal mortality. When it is very high, we observe exactly the opposite, represented almost in its entirety by the post-neonatal mortality^15^.

The age group that contributed the most to the decline in infant mortality was the early neonatal with an APC of -5.83, followed by the post-neonatal, with an APC of -3.7 in Rio Branco. The component with the lowest contribution was the late neonatal (-0.32).

A comparable situation occurred in Guarulhos, state of São Paulo, from 1996 to 2011, the APC of early neonatal mortality was -12.8 between 1996 and 2002 and remained stable until 2011. The post-neonatal component presented an APC of -5.7 for the whole period^16^.

Other studies have observed a higher concentration of deaths in the early neonatal component^3^ ^,^ ^17^, suggesting causes of deaths associated with prenatal care and delivery, inadequate attention to the newborn in the delivery room and the neonatal unit, requiring greater perinatal care^18–21^.

In the Federal District, from 1990 to 2000, the APC of the early neonatal component was -4.76 and the post-neonatal period was -7.80^22^. In Porto Alegre, from 1996 to 2008, the APC of the neonatal component was -3.5 and for the neonatal component, it was -4.1^23^.

Neonatal mortality rates also showed a significant decrease in Brazil between 1990 and 2007. The APC was -3.2, less pronounced than the overall decline in infant deaths. In contrast, post-neonatal mortality achieved an APC of -8.112.

Worldwide, the neonatal mortality rate has declined from 36/1,000 LB in 1990 to 19/1,000 LB by 2015. In Latin America and the Caribbean, in the same period, it decreased from 23/1,000 LB to 10/1,000 LB, and in developed countries from 8/1,000 LB to 4/1,000 LB^7^.

The groups of causes that most contributed to the infant deaths in Rio Branco in the period were those of perinatal conditions, followed by malformations, infectious and parasitic diseases, and respiratory diseases.

The Ministry of Health, when comparing infant deaths in Brazil in 2000, 2005, 2010 and 2012, observed that perinatal and maternal factors are the main causes in children under one year, corresponding to 52% of infant deaths in 2012, followed by congenital malformations responsible for 20.5% of infant deaths in 2012^24^.

In Aracajú, state of Sergipe, the main causes of death from 2001 to 2005 were also perinatal conditions with mean IMR of 18.1/1,000 LB with a significant reduction to 12.3 from 2006 to 2010. The causes of infant mortality due to congenital malformations and external causes increased in the periods^25^.

According to the survey “Birth in Brazil”, neonatal deaths occurred mainly due to prematurity, congenital malformation, and infections. The Northeast and North regions had the highest proportion of deaths due to infection (26.9% and 20.7%). The highest proportion of deaths due to congenital malformations occurred in the South and Southeast of Brazil^26^.

The reduction of mortality in Brazil is mainly due to the decline in preventable deaths^20^ ^,^ ^27^ ^,^ ^28^. According to a technical note from the Ministry of Health between 1996 and 2007, 70.0% of infant deaths could have been avoided^29^. An analysis of trends in infant deaths in Brazil between 1997 and 2006 estimated a 37.0% reduction in preventable causes, 75.7% in ill-defined causes and 2.2% in other causes of death^28^.

Mortality rates due to preventable causes had a reduction of 52.7% in Rio Branco between 1999 and 2015 (APC = -5.04) and poorly defined causes were reduced by 95.1% in the same period (APC = -10.89). The significant reduction in the proportion of deaths classified as ill-defined causes of death in Rio Branco suggests greater access to health services.

The quality of the information of a system can be evaluated according to its completeness. In the case of death information systems, when poorly defined, unreported or ignored causes are below 10%, the information is considered as of high quality^30^.

The mean frequency of ill-defined causes in Rio Branco in this study represented 6.6% of total infant deaths in the period, characterized as high-quality information. For the present analysis of the mortality trend from avoidable causes, it was assumed that ill-defined causes would have a proportional distribution similar to well-defined causes.

Despite the significant reduction of avoidable and ill-defined causes in Rio Branco, causes not clearly avoidable (other causes) did not show this trend. Because avoidable causes are sensitive to good quality health care, Rio Branco, like all of Brazil, may be experiencing improvement in health system performance. This hypothesis is supported by the notable improvement in the supply, coverage, and use of actions and services linked to SUS^8^ ^,^ ^24^ ^,^ ^28^. Malta et al.^28^ also point out that non-preventable causes tend to decline more slowly than preventable ones.

The main limitation of this study refers to the use of the direct method for the calculation of infant mortality since it depends on the coverage and quality of the basic data on births and deaths. The underreporting of deaths in the country is a problem to be faced, especially in the North and Northeast regions. This causes the Ministry of Health to recommend corrections for sites whose SINASC coverage is less than 90% or 80% of a composite index^1^. However, in recent publications of the Ministry of Health about the active search for deaths and births in the legal Amazon, the state of Acre presented the greatest coverage in the registry of deaths and births in the North. In addition, the coverage of Rio Branco capital resembles that of South and Southeast states, justifying the use of the direct method in this study.

The trend of infant mortality in Rio Branco follows the Brazilian scenario of reduction. The age component that contributed most to the decline in the infant mortality rate was the early neonatal, followed by the post-neonatal period. The late neonatal component contributed little to the reduction of infant mortality in the capital of Acre during the period analyzed.

The group of causes that most contributed to the infant deaths during the period was perinatal diseases, followed by malformations, infectious and parasitic diseases, and respiratory diseases.

With regard to avoidability, the IMR for avoidable causes showed a reduction of 46.3% between 1997 and 2013, the IMR for ill-defined causes decreased by 93.3% in the same period.

The significant reductions in ill-defined causes in Rio Branco suggest greater access to health services, with a possible improvement in health care. This allows the identification of the cause of death or improvement of completion in the death certificate and management of the SIM, seeking to better qualify the information available. However, infant mortality in the capital is relatively high compared to that of developed countries.
